# Authors’reply: Concern regarding the alleged spread of hypervirulent lymphogranuloma venereum *Chlamydia trachomatis* strain in Europe

**DOI:** 10.2807/1560-7917.ES.2017.22.15.30512

**Published:** 2017-04-13

**Authors:** Fruzsina Petrovay, Eszter Balla, Timea Erdősi

**Affiliations:** 1 Department of Bacteriology II., National Centre for Epidemiology, Budapest, Hungary; 2 Department of Phage and Molecular Typing, National Centre for Epidemiology, Budapest, Hungary

**Keywords:** Hungary, sexually transmitted infections, bacterial infections, lymphogranuloma venereum – LGV, *Chlamydia trachomatis*, men who have sex with men (MSM), genovariant

**To the editor:** A recent letter by Chlamydia researchers [1] reflected on our article [2], raising a question about a potential misclassification of the published Hungarian LGV genotypes that we characterised as ‘L2c’.

We agree with the authors of the letter that there is no established official nomenclature for lymphogranuloma venereum (LGV) genovariants. Moreover there are two so-called L2c variants reported in the literature, based on different typing methodologies [3,4]. The reference that we used to characterise the strains in our report was the L2c variant described by Somboonna et al. [4]. In particular, a partial sequence of the *ompA* gene of this variant was employed for typing, and, as stated in the letter, ‘*It is important to recognise that the use of the term ‘L2c genotype’ in the case of the L2-D recombinant strain is a misnomer, as the* ompA*-genotype of this strain is an archetypal L2’*, this sequence proved to be identical, at least at protein level, to the L2 sequence.

This fact was clarified by the LGV Genotype Dynamics Study Group, University of Basel. Due to the confusing situation of the LGV nomenclature, we have contacted this LGV research laboratory aiming to further collaborate and subtype the DNA-samples of the reported Hungarian LGV strains. We would like to point out that the Hungarian genovariants differ from the L2b variant spreading in western European countries, as was confirmed by comparison to different reference sequences of L2b sent by the LGV Genotype Dynamics Study Group, who suggested to describe the Hungarian isolates as ‘L2‘ genovariants until further more detailed genomic analysis.

As we do not know yet whether they prove to be a new L2 type or not, we recommend to wait with the classification of these strains until we have the final typing results. We agree that only further investigation, such as whole genomic sequencing and phylogenetic analysis can confirm the genomic background and these techniques may reveal some misnomers of LGV genotypes reported previously in other publications.
